# Rural South African mothers’ perspectives on strategies to mitigate cerebral palsy caregiving

**DOI:** 10.4102/ajod.v14i0.1632

**Published:** 2025-07-07

**Authors:** Ngokwana C. Rachamose

**Affiliations:** 1 Department of Psychology, Faculty of Humanities, University of the Witwatersrand, Johannesburg, South Africa

**Keywords:** caregiver, cerebral palsy, facilitator, rural, South Africa, support

## Abstract

**Background:**

Ample evidence suggests that primary caregivers of children with cerebral palsy experience barriers relating to their caregiving role; however, these caregivers also reported encountering factors that facilitate their caregiving experience.

**Objectives:**

This article aimed to explore factors that facilitate and support caregivers of children living with cerebral palsy in rural South Africa.

**Method:**

An exploratory qualitative research design was employed. Purposive convenience and snowball sampling were used to select 10 primary caregivers of children living with cerebral palsy between the ages of 3 years and 18 years. A semi-structured interview was used to collect data. Data were analysed using thematic analysis.

**Results:**

This research identified several factors for facilitating and supporting caregivers of children living with cerebral palsy. These include social support, caregivers’ ability to understand and accept their children’s disability, mental health support and caregivers’ access to resources.

**Conclusion:**

The study found that caregivers looking after children living with cerebral palsy in rural communities have access to certain support systems that aid their caregiving experiences; however, such systems of support need to be strengthened and sustained to reduce the burden of care.

**Contribution:**

This article highlights the facilitators and supportive factors of caring for children living with cerebral palsy in rural communities of South Africa to inform stakeholders on possible intervention strategies for maternal mental health in the context of raising a child with limiting disabilities.

## Introduction

Cerebral palsy (CP) describes a group of ongoing, activity-restricting abnormalities, mostly affecting mobility, muscle tone and postural control caused by an absence of typical progress in the brain of a growing foetus or newborn (Hallman-Cooper & Rocha Cabrero 2024). Movement abnormalities in individuals with CP are commonly in conjunction with sensory, perceptual, cognitive, communication and behavioural difficulties, along with epilepsy and bone and joint problems (Hallman-Cooper & Rocha Cabrero 2024). Cerebral palsy can manifest in a variety of ways, including a variable degree of presentation (spastic, athetoid, ataxic, hypotonic and mixed) and functionality (walking, communicating, moving and handling objects, eating, drinking and swallowing) (Proctor 2022). This indicates that some children may require minimal assistance with functionality, while others may require more assistance or rely entirely on the caregiver for daily functioning (Paulson & Vargus-Adams 2017). These challenges may lead to a series of psychological problems and even cause chronic sorrow in the primary caregiver, characterised by feelings of disappointment, shame, hopelessness, frustration and exhaustion (Nimbalkar et al. 2014). Further, caregivers raising children living with CP are exposed to psychosocial problems, including family conflicts, lack of sufficient finances and resources, insufficient information, and experiences of stigma and marginalisation, all of which may affect the family’s quality of life and overall functioning (Fairfax et al. 2019; Rachamose & Harvey 2024).

Although many previous studies have reported several barriers including financial, psychological, social, health and environmental obstacles among the parents of children with CP (Abdullahi & Isah 2020; Cooper et al. 2024; Pretorius & Steadman 2018; Rachamose & Harvey 2025), some recent studies have revealed positive and promising attitudes in these parents (Alibakhshi et al. 2021). Additionally, it has been demonstrated that a positive attitude, financial aid, religion and adequate support systems are all factors that facilitate the caregiving of children with CP (Alibakhshi et al. 2021; Davis et al. 2010; McManus et al. 2006; Pretorius & Steadman 2018; Thrush & Hyder 2014). In other words, certain caregivers have successfully overcome obstacles associated with caring for their children living with CP and returned to ‘normal’ family life with an appropriate support system in place. For instance, in Ghana, a study discovered that caregivers depend on social support, including family, neighbours, friends, co-workers and community members, as well as governmental and non-governmental initiatives, to alleviate the burden of providing care and to accomplish their caregiving goals with children living with CP (Amin Sayed, Hamdy Abdelmonem & Ali Ahmed 2021). Further, community and caregiver educational programmes have been shown to reduce the burden of caregiving through empowering caregivers (Prieto 2020). Similarly, non-profit organisations (non-governmental organisations [NGOs]) have been found to offer a supportive environment for caregivers of children living with CP in low-resource rural settings in South Africa (SA), Lesotho, Uganda and Malawi (Bray et al. 2017). Non-profit organisations have been found to also foster understanding, optimism, assurance, practical abilities and enhanced familial connections, thereby alleviating feelings of isolation and self-blame (Bray et al. 2017). Nevertheless, research conducted in rural Limpopo has revealed that caregivers of children with CP are burdened with a significant amount of care because of the limited resources, services and support available to them from both governmental and non-governmental organisations (Sodi & Kgopa 2016). This is despite the fact that their experiences have not been well documented. Consequently, the purpose of this article is to explore the facilitators and supportive factors available to caregivers of children living with CP in rural Limpopo, SA. A facilitator denotes any element that makes caregiving more manageable (Stevenson 2010).

### Theoretical framework

The Ecological Systems Theory (EST) (Bronfenbrenner 1977) was used as a theoretical framework for interpreting and understanding the findings. The EST suggests that strategies to mitigate the challenges encountered by caregivers of children living with CP are influenced by multiple interconnected layers that may foster resilience under challenging circumstances; these include the microsystem (immediate environment), mesosystem (connections between environments), macrosystem (social and cultural values), exosystem (indirect environments) and chronosystem (environmental changes over time).

## Research methods and design

### Study design

A qualitative exploratory contextual research design was used to gain a more in-depth comprehension of primary caregivers’ experiences of raising a child living with CP in rural communities of the Limpopo province of SA. Semi-structured, one-on-one interviews were conducted to explore and elicit as much information about the caregivers’ experiences, mental health, barriers and facilitators to caregiving.

### Study setting

The study was conducted in three districts (Capricorn, Sekhukhune and Waterberg) of the Limpopo province, SA. Limpopo is one of SA’s most rural provinces, with almost 80 per cent of the population residing in rural regions or villages. Research demonstrates that rural parts of the Limpopo province have a greater unemployment rate; people tend to work for lower salaries, have lower levels of education, have a greater percentage of low-income families, and rely heavily on government social grants (such as the old age and child grants) to make ends meet (Malatji 2020). This situation presents tremendous socio-economic obstacles that may affect the well-being of the primary caregivers of children living with CP, making it more important to explore their experiences.

### Study population

The study population comprised maternal primary caregivers (these included biological mothers, grandmothers, aunts or legal parents who include adoptive or foster mothers who take care of their children living with CP on a full-time basis) as eligible participants; however, only the biological mothers participated. This approach aimed to ensure that the study accurately captured the experiences of individuals who are actively involved in the daily care of children living with CP. Further, eligible maternal primary caregivers were above the age of 18 years to ensure that they were of consenting age. The children of the participants were between the ages of 3 years and 18 years of age to encourage the possibility that the caregivers will have some experience to draw on in their interviews, as opposed to caregivers of newborns. Further, the children had to be diagnosed with CP as the primary diagnosis, and the diagnosis had to be made by a medical professional such as a general practitioner or paediatrician. Finally, the children had to live with the participant, and the participant had to be the primary caregiver of the child residing in the rural community (Capricorn, Sekhukhune and Waterberg) of the Limpopo province. There was a total of 10 maternal primary caregivers (all of whom were biological mothers to the children) who were eligible for the study and consented to participate. The maternal primary caregivers in the three districts were selected to highlight the distinct socio-economic and cultural aspects that affect caregiving for a child living with CP. The inclusion criteria allowed direct insights of those involved in everyday caregiving, providing insight into both the barriers and facilitators. The use of purposive convenience and snowball sampling until data saturation allowed for rich, in-depth data exploration, strengthening the study’s rigour.

### Data collection

Data were collected from consenting maternal primary caregivers using semi-structured, one-on-one interviews. The author developed an interview schedule after reviewing relevant literature on the experiences of caring for a child with CP in rural communities. The interview schedule included core questions as well as clarifying prompts. For instance, Question 2 in Box 1 (also see Appendix 1 for the interview schedule) asks about caregivers’ overall health, accompanied by three probing questions about their physical, mental, and emotional health.

BOX 1 Core questions from one-on-one interviews.

**Questions:**

How does raising your child in a rural area specifically impact your role as a maternal caregiver of a child living with CP?

What access do you have to support services for raising your child living with CP?

How is your overall health?

Physically, mentally and emotionally?

What kind of professional assistance for yourself, if any, are you regularly receiving?

Can you elaborate on this?

What do you think can be (further) done to help you raise your child?

What do you think can be done to make things easier for you?

Note: see Appendix 1 for the interview schedule.

CP, cerebral palsy.

Before data collection, Clare Harvey (CH) reviewed the interview schedule to ensure that the questions were clear, understandable and capable of answering the research questions. Furthermore, the first interview served as a pilot interview; however, no adjustments were made to the interview schedule. All interviews were conducted over the cellphone with consent to record. The author purchased airtime and contacted each participant on their respective cellphones. Each interview lasted 45 min – 60 min, at a time that was convenient for each participant. The interviews were conducted in the participants’ preferred languages (primarily Sepedi and English), transcribed verbatim, and then translated into English by the author. The author conducted the interviews, transcribed both the English and Sepedi interviews, and translated the Sepedi interviews. A colleague fluent in Sepedi signed the data-sharing contract to facilitate back translation, ensuring the accuracy of the transcription and translation. Data collection took place between December 2023 and January 2024.

### Data analysis

Data were analysed using a six-phase process thematic analysis (Braun & Clarke 2019). Thematic analysis enabled the author to methodically code, categorise and examine large amounts of data. The author used both the inductive and deductive methods of analysis. This was done by analysing data with respect to the themes from the study’s literature review or the research questions. However, any noteworthy or relevant data (themes) that appeared from the transcriptions were also analysed, including unexpected themes, to gain an understanding of the primary caregivers’ experiences. The author categorised the data, which was then checked by CH. The author and CH conducted an additional abstraction of the data to facilitate the development of themes that are pertinent to the study’s objective.

### Ethical considerations

Before conducting the interviews, an application for full ethical approval was made to the University of the Witwatersrand Human Research Ethics Committee (Medical). The ethics approval number is M230803 M231023-B-0001. Ethical standards of informed consent, confidentiality and management of information were adhered to.

## Results

Table 1 illustrates the demographics of the caregivers interviewed in this study. The biological mothers’ ages range from 34 years to 45 years, with a median age of 40 years. Of the caregivers, 50% are single and are unaware of their child’s diagnosis type, while 40% are unemployed.

**TABLE 1:** Demographics of the caregivers interviewed in this study.

Participant pseudonym	Age (years)	Relationship to the child	Relationship status	Employment status	Child’s age (years)	Diagnosis type
Anna	36	Mother	Married	Self-employed	13	Unknown to the mother
Eve	45	Mother	Married	Employed	5	Spastic quadriplegia CP
Diana	36	Mother	Single	Unemployed (Volunteering)	14	Spastic CP
Elisa	41	Mother	Married	Self-employed	7	Dyskinetic CP
Maria	40	Mother	Single	Unemployed (Volunteering)	15	Choreoathetoid CP
Johana	45	Mother	Single	Self-employed	8	Unknown to the mother
Esther	39	Mother	Single	Unemployed	6	Unknown to the mother
Abigail	34	Mother	Married	Employed	9	Unknown to the mother
Ruth	42	Mother	Married, Separated	Unemployed	3	Unknown to the mother
Magdeline	38	Mother	Married	Self-employed	10	Spastic diplegia CP

*Source:* Adapted from Rachamose, N. & Harvey, C., 2025, ‘The barriers to caring for a child living with Cerebral Palsy (CP) in rural Limpopo, South Africa’, *Disabilities* 5(1), 11. https://doi.org/10.3390/disabilities5010011

CP, cerebral palsy.

The results of this study are summarised in Table 2.

**TABLE 2:** Themes and sub-themes.

Theme	Sub-theme
1. Social support	Spouse or partner’s support
	Support from family and friends
	Support from other mothers of children living with CP
	Community and NGO support
	Medical practitioners’ support
2. Understanding and accepting the child’s disability	-
3. Mental health support	-
4. Caregivers’ access to resources	-

CP, cerebral palsy; NGO, non-governmental organisation.

### Social support

Many caregivers reported having one or more social support systems to help them cope with raising their children living with CP. These support systems included family, friends, other mothers of children with CP, community members including NGOs, and medical professionals.

#### Spousal or partner’s support

All caregivers in a relationship reported that their partners were incredibly supportive as co-caregivers. Eve stated that her unemployed husband is the primary caregiver during the day because she is employed. Some caregivers stated that their spouses were a valuable source of income, alleviating the financial burden or compensating for their inability to work:

‘Well, I wake up, I bath I go to work, then the child will remain with father and other children because my husband does not work, so he knows him very well and he knows his routine, then I will see him later when I come back from work.’ (Eve, P2, 45)

‘I got another one [wheelchair] from Dr Luke at Mediclinic … it was R16 000 … it was expensive but used a medical aid…medical aid is being paid by her father.’ (Elisa, P4, 41)

#### Support from family and friends

Caregivers also expressed that receiving assistance from their family and friends aided them in caring for their children. According to their report, friends and family provided significant support during the birth of their children. In addition, the family and friends of the caregivers provided valuable learning experiences and advised them to embrace the circumstances rather than reject their child. The family’s acceptance and love for the child also helped to decrease stress levels:

‘My children help me, my siblings help, my mom helps even though she doesn’t have the patience, my children know him [*the child living with CP*], so they help feed him, bath him, and they play with him and keep him active, he is never alone, he also likes TV, we all watch cartoons with him, all my kids are okay, we are no longer stressed, we have accepted.’ (Maria, P5, 40)

‘The friends we have now, they treat her like their own kids, they are caring and loving, and they understand the circumstances.’ (Elisa, P4, 41)

#### Support from other caregivers of children with cerebral palsy

Caregivers also recounted getting support from other caregivers in similar circumstances. Some caregivers met during hospital visits, while others met through non-governmental organisation (NGO) initiatives, and this allowed them to form support groups where they could share their experiences, and advice, comfort one another, and share information about resources such as wheelchairs and standing frames. Caregivers remarked that this support network helped them feel supported and not alone, despite the fact that some of them are separated by distance:

‘The people who support me are the mothers at the hospital, they helped me with wheelchairs, my child did not have a wheelchair before, they helped get one. They also helped me get the standing frame so that when the child is tired of sitting on the wheelchair, she can use it. They are the ones who support me because they know lot of things.’ (Abigail, P8, 34)

‘Yes, even the [*NGO*] helped, we are a group of twenty, twenty parents of children with CP, so having those mothers in a group was helpful.’ (Maria, P5, 40)

#### Community and non-governmental organisation support

According to the caregivers, an NGO provided tremendous support in the Sekhukhune district. Caregivers reported that the NGO delivered workshops that empowered them and their families, particularly about CP and how to better understand and care for their children. In other districts, caregivers emphasised the importance of the community, including neighbours and churches, in accepting and supporting their children. Diana stated that she received a wheelchair from the municipality, which helped her carry her child, a change from using a wheelbarrow:

‘In 2014, [*an NGO*] came to Dilokong Hospital … I attended the workshop for three weeks, they taught me what is CP, different levels and types, what is chorea, what is spastic, CP as a way of life, that is why when I came back from that workshop, I came back full of life, it’s like God had restored things, they helped me a lot, the manuals, the pictures. When I got home, I was able to workshop my family especially on spastic because we had a spastic CP at home. They had a better understanding on how to hold, how to lift, how to go around a spastic CP child. That workshop empowered me.’ (Diana, P3, 36)

‘I was able to access resources even in the rural [*area*] because I am not ashamed to talk about my child when I am with strangers. The municipality has donated a proper wheelchair, and the hospital also gave him the wheelchair, so we do push him when we go to shops so he can see people. It’s obviously difficult raising a child in a rural area but it didn’t really impact me because I was able to access those resources … the wheelchair was helpful because my older son used to carry him with a wheelbarrow, now he can use the wheelchair.’ (Ruth, P9, 42)

#### Medical practitioners’ support

Other caregivers reported that medical practitioners also served as facilitators. Eve described how medical practitioners in her rural clinic collaborate with those in town or a tertiary hospital to provide advanced medical care. Other caregivers noted that the physiotherapy department at the local hospital offered them education about CP, including clarification on the myths about CP:

‘From the physio department, they taught us what is CP, how it works, how to accept, they counselled us and even explained that it is not witchcraft.’ (Abigail, P8, 34)

‘Yes, they [*local nurses and doctors*] take us to better hospitals in town when they think there is a need. I have travelled many times with other parents to those hospitals where their kids need different special treatment, and they received them … That is why I do not think rural or town matters, they can take these kids to those towns and help us.’ (Eve, P2, 45)

Furthermore, caregivers reported an interest in being active in their child’s disability management. Diana contended to understand more about her child’s CP’s presentation than medical professionals, and as a result, she expressed the need to be involved in her child’s treatment. She emphasised the need to be involved in her child’s physiotherapy training:

‘For me, it is involvement and participation in my child’s care, because I know the child better than the doctor, the doctor only sees the child for an hour, but I see him for 31 days, I wish there could be a doctor for speech, to help improve my child speech, for example normal kids they easily know mama and papa, and the rest you can teach them later, but a child with CP you don’t even know where to start to improve their speech. I can see my child is trying to talk but it’s not easy, and I am also not trained to teach my child speech. I need that skill. Because most of the children with CP need to be trained on toileting, and speaking, even though they cannot really explain well, as long as the mother can understand what the child is trying to say.’ (Diana, P3, 36)

### Understanding and accepting their child’s cerebral palsy

Understanding and accepting their child’s CP was one of the facilitators mentioned by the primary caregivers. Many caregivers attributed their understanding and acceptance of their children’s CP to being exposed to similar children and their children’s better health conditions in comparison to other children with similar disabilities. Caregivers expressed that seeing other children with disabilities inspired them to continue caring for their own. Other caregivers stated that interacting with other children with disabilities at the hospital helped them accept their children’s disability. Many caregivers emphasised the importance of seeing other children in hospitals, including those with hydrocephalus and other limiting disabilities, to understand that they are not alone in experiencing the disability:

‘I think going to other hospitals and seeing other children in the big hospitals … You see that it’s not just your child, and maybe your child is better. You even see worse conditions, some with hydrocephalus, some who can’t do anything, you will see that you are just having too much stress for nothing, and these kids are now many, and it’s not just our children, even white and Indian kids I have also seen, so seeing that there are other children, and I am not alone.’ (Maria, P5, 40)

‘I would go to the hospital and I saw other children and I started accepting because I could see that I was not the only one, because where I stayed you hardly see such things or any child with disabilities, I had never seen a child with disabilities, she was the first I saw and the ones I saw at the hospital.’ (Abigail, P8, 34)

Several caregivers also stated that accepting their children’s disability helped them feel better. They highlighted that, while it took them some time to accept, and others are still working on it, many caregivers have noticed an improvement in their mental health as a result of accepting their children’s CP. They explained that they are gradually learning to live like other people who do not have children with disabilities:

‘At first, it was so hard that I even felt like the world could just open and I enter, but since I have managed to accept, life has moved on, there is no problem, I live like everyone, even like those who do not have children with CP, I sometimes even look better than them.’ (Maria, P5, 40)

‘I realised others love their children, even saw on Facebook where they post them and show them love and I was motivated to also accept mine and give her motherly love.’ (Johanna, P6, 45)

### Mental health support

Many caregivers expressed the need for mental health support. Caregivers acknowledged a need for counselling to manage difficult and frustrating feelings and emotions while caregiving:

‘Eish, I don’t know, the biggest thing that bothers me is my heart, I am complaining a lot, I am thinking a lot, I don’t know what they can do to help me.’ (Eve, P2, 45)

‘Please I really need counselling from time to time because somethings I would like to talk to someone to navigate some things that I can’t really talk to anyone with.’ (Abigail, P8, 34)

‘Yes, people talk, but most times things are not directly said to me, but they get to me, but I just ignore them, you will not even see if I am not okay, but I am hurting, and I just need someone to talk who can understand me.’ (Johanna, P6, 45)

#### Caregivers’ access to resources

Caregivers also identified the need for resource assistance as an additional support strategy. Caregivers expressed a need for a monthly supply of nappies or diapers and mobility aids, including wheelchairs, standing frames and special food items, to alleviate the financial burden. They also requested specialised schools to allow them a respite from full time care of their child with CP:

‘Things like a wheelchair, we need wheelchairs to support, to support our children, especially for kids my age and with this type of CP, we recently saw this wheelchair that you can feed, bath and sleep your child on, but it is expensive. Now he is 15, next year he is turning 16, he must go and make his ID, he needs a wheelchair … some other things we can raise them but they will say we are unreasonable, things like pampers, they will tell you that you get SASSA [*South African Social Security Agency*] for this child. He is old, I am buying size 6, and … that six is R200 and they are 28 in number, and I have to buy 2 from R400, then I have to buy special food like Nespray Milk, Weet-bix, soft food, they are picky these children.’ (Maria, P5, 40)

‘I would be happy if we had schools that would accommodate our children. My child is intellectually fine, she is just paralysed on the legs, she can speak, she understands, and she can’t walk, but I think she can learn like other normal children, I just need a special school that can accommodate her because there is no point of her staying at home while she is able to learn like other children. I can even give her my phone and you speak to her; you will hear that she is well intellectually. So, I need a school.’ (Magdaline, P10, 38)

‘It’s definitely school, I have walked around looking for a school. They all ask me the same question, can she take herself to the bathroom, and she can’t, so they decline her … What I need, I wish I could find schools for children with CP around this area, especially for children with similar conditions to mine … I think what will be great to assist me, is if she can get a special school where she can attend a few hours a day so that she can get stimulated, and have peers over.’ (Abigail, P8, 34)

## Discussion

Research shows that while caring for children living with CP comes with many obstacles including physical, financial, social, emotional, spiritual and health barriers (Dlamini, Chang & Nguyen 2023; Eyong, Ekanem & Asindi 2017; Madzhie et al. 2022; Rachamose & Harvey 2024; Singogo, Mweshi & Rhoda 2015; Smith & Blamires 2022), caregivers also have facilitators that make their caregiving processes bearable (Alibakhshi et al. 2021; Cooper et al. 2024; Pfeifer et al. 2014; Pretorius & Steadman 2018; Raphulu et al. 2021). The EST provides a theoretical framework for explaining and understanding the barriers and facilitators of caring for a child with CP by exploring caregiving across multiple interconnected levels of influence, such as micro, meso, macro, exo and chrono systems (Bronfenbrenner 1977). These include social support from broader social contexts as well as caregivers’ understanding and acceptance of their children’s CP. Caregivers in this study also proposed additional facilitators and supportive factors that can make their lives and that of their children better, and these include mental health support and caregiver resources provision.

The findings of this study are consistent with previous research which has demonstrated that at the microsystem which encompasses the immediate environment of caregivers (family, close friends and neighbours), social support can be a protective factor during stressful events and can be a significant predictor of parental adjustment in the face of stress associated with the birth of a disabled child (Pfeifer et al. 2014). This was shown in this study, where many caregivers who had some form of support from either friends, family and/or community reported adjusting well to their caregiving role compared to those who reported having no one who expressed feeling isolated, helpless and alone (Rachamose & Harvey 2024). This suggests that social support assists caregivers to understand and care for those with disabilities in helpful ways (Phumudzo, ShirindI & Makofane 2021). They help to transform a sense of hopelessness, isolation and guilt into pride, acceptance and increased self-confidence (Saloojee & Bezuidenhout 2020). Many mothers also reported appreciating any form of support received, even though most support was mainly provided by those from the core family (husband, mother, siblings, children) (Milbrath et al. 2008). This shows that caregivers consider the quality of support as more important than the number of people who give this support (Pfeifer et al. 2014).

At the mesosystem that denotes interactions between microsystems, such as interactions between the caregiver and health institutions, understanding and accepting their child’s CP in this study played a huge role as a facilitator in caring for a child living with CP. The overall well-being of caregivers in this study was reported to improve when they understood their children’s diagnosis and when they saw that they were not the only ones with children with CP. A finding similar to that of Guillamón et al. (2013) and Rachamose and Harvey (2024) who found that after the diagnosis, parents seem to be more eager for information related to the causes of the disorder and possible impairments, therapies and prognostics. Further, when caregivers were clear about their children’s diagnosis, they reported having a positive attitude towards their children’s disabilities. This finding aligns with that of Alibakhshi et al. (2021), which showed that positive attitudes and beliefs are factors responsible for promoting acceptance in caregivers of children with CP. Positive beliefs seem to be effective in reducing stress, creating resilience and improving parents’ mental health in the case of raising a child living with a disability (Mbatha & Mokwena 2023). Spiritual beliefs as part of the macrosystem, which includes broader cultural values, beliefs and ideologies, were prevalent in this study. It is important to note, however, that spiritual beliefs in this study were both a barrier and a facilitator at the same time. Many caregivers believed that it was God’s will that they had children with CP and had hopes that God would heal their children; however, it also seemed that they felt they were being punished, a finding similar to existing research (Rachamose & Harvey 2024). A finding similar to that of Chataika (2013) shows that certain African nations continue to see disability as a punishment inflicted by ancestral spirits dissatisfied with a family or person, or as a consequence of witchcraft, where grievances must be addressed. Although beneficial at times, these beliefs also resulted in the stigmatisation of primary caregivers of children with disabilities and their families (Pretorius & Steadman 2018) such that caregivers will be unwilling to form social relationships because of decreased mood and problems faced in the social and professional spheres (Pfeifer et al. 2014).

Within the exosystem, encompassing environments that indirectly affect the individual, such as access to healthcare or resources, mental health support was identified as one of the proposed facilitators by the primary caregivers in this study. A finding also identified by Guimarães et al. (2023) states that one of the parental challenges of caring for children with CP is the internal challenge of mental health related to increased risk of experiencing high stress, anxiety and depression (Rachamose & Harvey 2024). As such, parents get involved in crucial needs of information, support and personal well-being services available in the community, such as rehabilitation centres and emotional and/or physical growth and development (Buran et al. 2009). Many caregivers in the present study reported not knowing where to access mental health services, they only remember receiving one-time counselling services during their children’s diagnosis. This is because access to mental health services remains an international challenge, especially in rural and underserved areas (Bray et al. 2017; Saloojee & Bezuidenhout 2020). Consequently, the majority of caregivers of children with disabilities have limited access to interventions that promote mental well-being (Iemmi et al. 2016). Regardless, the present study showed that few caregivers received psychological support from community-based rehabilitation and have reported a significant turnaround in their mental health as a result. Previous literature has shown that community-based rehabilitation, such as parent-to-parent peer support for parents of children with disabilities, has been found to improve emotional and psychosocial well-being (Bray et al. 2017). Consequently, it is imperative to implement and provide support for mental health initiatives that are centred around the family and community. Additionally, healthcare professionals must plan and be proactive in providing medical and social support for children living with CP and their families (Liddle et al. 2018).

Finally, at the chronosystem, that is, the broader society and policy level, caregivers proposed the provision of resources such as finances and specialised schools as facilitators to caring for their children. Previous studies have shown that in Africa, poverty significantly impeded the ability of caregivers to provide basic needs for their children with disabilities (Donald et al. 2014; Pretorius & Steadman 2018). The huge expenses of medical treatment, therapy, assistive devices and transportation significantly contributed to caregivers’ lack of access to care (Rachamose & Harvey 2024). As a result, caregivers benefited from several social policies that had been implemented to address inequality among individuals living with disabilities (Pretorius & Steadman 2018). In an effort to enhance healthcare accessibility, the South African government implemented a policy that ensures free healthcare services to pensioners, expectant women, individuals living with disabilities and children under the age of 6 years (Barratt & Penn 2009). Additionally, individuals with special needs have been provided access to grants (Barratt & Penn 2009). Despite government financial assistance often being the only source of income and being inadequate to meet the family’s needs, caregivers considered it one of the most significant facilitators of caregiving (Pretorius & Steadman 2018). Therefore, the provision of specialised CP schools, food and financial aid may mitigate the challenges arising from the scarcity of resources.

A more effective approach to support these caregivers may include facilitating their engagement in income-generating activities by providing them with time. The provision of respite services and the construction of daycare facilities and special schooling for children with disabilities may provide caregivers more time to seek employment (Pretorius & Steadman 2018). The literature underscores the need for respite programmes as caregivers often experience relief from their duties only when their child is at a care facility or school for extended hours every day or week (Pizano-Vega et al. 2020). While most of the caregivers in this study had difficulties obtaining sufficient respite services, a few successfully located a suitable care facility for their child within their neighbourhood. The caregivers acknowledged the significance of their child’s crèche, school or daycare as they appreciated the time off from their caregiving responsibilities that these institutions provided (Ayob, Christopher & Naidoo 2021).

### Limitations and recommendations

The main limitation of this study is the inclusion of caregivers exclusively from one province, specifically, biological mothers. This study is limited to a single perspective despite the provision of valuable insights regarding location-specific facilitators and supportive factors. Therefore, future studies should focus on assessing the effectiveness of mental health interventions, financial assistance programmes and NGO support initiatives for caregivers. Additionally, examining how policies can more effectively assist caregivers, particularly regarding access to CP-specialised education, healthcare and respite services, may enhance government and NGO strategies.

## Conclusion

This study demonstrated that the caregiving of a child living with CP in Limpopo comes with numerous obstacles, including inadequate access to essential resources. Nevertheless, caregivers in this study reported that they were able to benefit from supportive and facilitative factors, including family, friends, community members, NGOs and medical practitioners. These individuals played a critical role in educating, supporting and empowering them. This suggests the need to strengthen facilitators and supportive factors available for caregivers. This can be accomplished by involving all family members in the child’s care management alongside the mother. Additionally, efforts may be increased to enhance community-level awareness of neurological disabilities such as CP, ensuring a broader social understanding of CP and how community members can contribute to the well-being of primary caregivers. In addition, NGOs that provide support to primary caregivers of children with limiting disabilities must receive financial support to extend their services into various rural regions, as caregivers who had their support reported much more understanding of the CP than those without any NGO presence. Furthermore, caregivers identified the need to access continuous mental health care and CP-specialised schools in rural areas. Mental health and respite services should be prioritised to alleviate the psychological burden of caregiving and provide relief to caregivers.

## Appendix 1: Interview schedule

1. What is your age?
2. Do you work, and if so, what do you do?
3. Who do you live with?
4. How old is your child with cerebral palsy?
5. What is your child’s gender?
6. Do you have any other children?
  - Ages?
7. What was your pregnancy and birth like with your child living with CP?
  - How was this for you emotionally?
8. When was your child diagnosed with CP?
  - Can you tell me about this process, including how this diagnosis came about?
  - What do you remember about this time, including how you felt?
9. What do you understand about CP?
  - How did you learn about this?
10. What was it like finding out about your child’s disability?
11. Who did you talk to after finding out about your child’s disability?
  - What was this like for you?
  - What were other people’s responses towards you and your child?
  - How did you manage these feelings and reactions?
12. How is the relationship like between you and your child?
13. What is it like raising your child, specifically with regards to their CP?
14. What does your day-to-day routine look like?
15. How has your child’s disability impacted your life?
  - Physically, mentally, emotionally, socially?
16. What are the challenges you face raising your child living with CP?
17. Who is helping you raise your child, if anyone?
  - What is helpful, unhelpful about this?
18. What other kinds of support systems do you have?
  - Can you elaborate on these? How did these come about? What is helpful about them?
19. What motivates you to keep looking after your child?
20. How does raising your child in a rural area, specifically, impact your role as a maternal caregiver of a child with a disability, specifically, CP?
  - What access do you have for support services with regards to raising your child living with CP?
21. How is your overall health?
  - Physically, mentally and emotionally?
22. What kind of professional assistance for yourself, if any, are you regularly receiving?
  - Can you elaborate on this.
23. What do you think can be (further) done to help you raise your child?
24. What do you think can be done to make things easier for you?
25. Is there anything else you would like to share that I may not have asked?
26. Do you have any questions?
27. Are you feeling okay to finish the interview now?

## References

[CIT0001] Abdullahi, A. & Isah, A., 2020, ‘Caregiver’s perspectives on facilitators and barriers of active participation in cerebral palsy rehabilitation in North West Nigeria: A qualitative study’, *BMC Health Services Research* 20, 615. 10.1186/s12913-020-05487-w 32631316 PMC7336653

[CIT0002] Alibakhshi, H., Azkhosh, M., Bahmani, B., Khanjani, M.S. & Shahboulaghi, F.M., 2021, ‘Hope facilitators in parents with children suffering from cerebral palsy: A qualitative study’, *Iranian Journal of Psychiatry and Behavioral Sciences* 15(2), e107430. 10.5812/ijpbs.107430

[CIT0003] Amin Sayed, M., Hamdy Abdelmonem, H. & Ali Ahmed, F., 2021, ‘Effect of empowerment program for caregivers on quality life of children with cerebral palsy’, *Egyptian Journal of Health Care* 12(1), 140–155. 10.21608/ejhc.2021.138611

[CIT0004] Ayob, Z., Christopher, C. & Naidoo, D., 2021, ‘Caregivers’ perception of their role in early childhood development and stimulation programmes in the early childhood development phase within a sub-Saharan African context: An integrative review’, *South African Journal of Occupational Therapy* 51(3), 84. 10.17159/2310-3833/2021/vol51n3a10

[CIT0005] Barratt, J. & Penn, C., 2009, ‘Listening to the voices of disability: Experiences of caring for children with cerebral palsy in a rural South African setting’, in M. MacLachlan & L. Swartz (eds.), *Disability and international development: Towards inclusive global health*, pp. 191–212, Springer, New York, NY.

[CIT0006] Braun, V. & Clarke, V., 2019, ‘Reflecting on reflexive thematic analysis’, *Qualitative Research in Sport, Exercise and Health* 11, 589–597. 10.1080/2159676X.2019.1628806

[CIT0007] Bray, L., Carter, B., Sanders, C., Blake, L. & keegan, K., 2017, ‘Parent-to-parent peer support for parents of children with a disability: A mixed method study’, *Patient Education and Counseling* 100(8), 1537–1543. 10.1016/j.pec.2017.03.004 28284463

[CIT0008] Bronfenbrenner, U., 1977, ‘Toward an experimental ecology of human development’, *American Psychologist* 32(7), 513–531. 10.1037//0003-066X.32.7.513

[CIT0009] Buran, C.F., Sawin, K., Grayson, P. & Criss, S., 2009, ‘Family needs assessment in cerebral palsy clinic’, *Journal for Specialists in pediatric Nursing* 14(2), 86–93. 10.1111/j.1744-6155.2008.00176.x 19356202

[CIT0010] Chataika, T., 2013, ‘Cultural and religious explanations of disability and promoting inclusive communities in Southern Africa’, *Dignity* 15, 117.

[CIT0011] Cooper, K., Crozier, K., Blair, S., Mcdermott, R. & Croal, I., 2024, ‘Exploring the barriers and facilitators to caregiver engagement in postural management training for children with complex neurodisabilities: Qualitative study’, *Physiotherapy* 123, e215. 10.1016/j.physio.2024.04.269

[CIT0012] Davis, E., Shelly, A., Waters, E., Boyd, R., Cook, K., Davern, M. et al., 2010, ‘The impact of caring for a child with cerebral palsy: Quality of life for mothers and fathers’, *Child: Care, Health and Development* 36(1), 63–73. 10.1111/j.1365-2214.2009.00989.x 19702639

[CIT0013] Dlamini, M.D., Chang, Y.-J. & Nguyen, T.T.B., 2023, ‘Caregivers’ experiences of having a child with cerebral palsy. A meta-synthesis’, *Journal of Pediatric Nursing* 73, 157–168. 10.1016/j.pedn.2023.08.026 37690430

[CIT0014] Donald, K.A., Samia, P., Kakooza-Mwesige, A. & Bearden, D., 2014, ‘Pediatric cerebral palsy in Africa: A systematic review’, *Seminars in Pediatric Neurology* 21(1), 30–35. 24655402 10.1016/j.spen.2014.01.001

[CIT0015] Eyong, K.I., Ekanem, E. & Asindi, A., 2017, ‘Challenges of care givers of children with cerebral palsy in a developing country’, *International Journal of Contemporary Pediatrics* 4(4), 1128–1131. 10.18203/2349-3291.ijcp20172656

[CIT0016] Fairfax, A., Brehaut, J., Colman, I., Sikora, l., Kazakova, A., Chakraborty, P. et al., 2019, ‘A systematic review of the association between coping strategies and quality of life among caregivers of children with chronic illness and/or disability’, *BMC Pediatrics* 19, 1–16. 10.1186/s12887-019-1587-3 31262261 PMC6600882

[CIT0017] Guillamón, N., Nieto, R., Pousada, M., Redolar, D., Muñoz, E., Hernández, E. et al., 2013, ‘Quality of life and mental health among parents of children with cerebral palsy: The influence of self-efficacy and coping strategies’, *Journal of Clinical Nursing* 22(11–12), 1579–1590. 10.1111/jocn.12124 23461414

[CIT0018] Guimarães, A., Pereira, A., Oliveira, A., Lopes, S., Nunes, A., Zanatta, C. et al., 2023, ‘Parenting in cerebral palsy: Understanding the perceived challenges and needs faced by parents of elementary school children’, *International Journal of Environmental Research and Public Health* 20(5), 3811. 10.3390/ijerph20053811 36900819 PMC10001820

[CIT0019] Hallman-Cooper, J.L. & Rocha cabrero, F., 2024, *Cerebral palsy*, StatPearls Publishing LLC, Treasure Island, FL. 30844174

[CIT0020] Iemmi, V., Blanchet, K., Gibson, L.J., Kumar, K.S., Rath, S., Hartley, S. et al., 2016, ‘Community-based rehabilitation for people with physical and mental disabilities in low-and middle-income countries: A systematic review and meta-analysis’, *Journal of Development Effectiveness* 8, 368–387. 10.1080/19439342.2016.1157623

[CIT0021] Liddle, M., Birkett, K., Bonjour, A. & Risma, K., 2018, ‘A collaborative approach to improving health care for children with developmental disabilities’, *Pediatrics* 142(6), e20181136. 10.1542/peds.2018-1136 30385639

[CIT0022] Madzhie, M., Mphephu, K., Baloyi, V. & Chueng, M., 2022, ‘The challenges experienced by mothers with children suffering from cerebral palsy: A study conducted at Mutale Municipality, South Africa’, *Cogent Psychology* 9(1), 2043020. 10.1080/23311908.2022.2043020

[CIT0023] Malatji, M.T., 2020, *Rural development outcomes and policies in South Africa’s Limpopo province*, Doctoral dissertation, University of South Africa, Pretoria.

[CIT0024] Mbatha, N.L. & Mokwena, K.E., 2023, ‘Parental stress in raising a child with developmental disabilities in a rural community in South Africa’, *International Journal of Environmental Research and Public Health* 20(5), 3969. 10.3390/ijerph20053969 36900985 PMC10001439

[CIT0025] Mcmanus, V., Michelsen, S.I., Parkinson, K., Colver, A., Beckung, E., Pez, O. et al., 2006, ‘Discussion groups with parents of children with cerebral palsy in Europe designed to assist development of a relevant measure of environment’, *Child: Care, Health and Development* 32(2), 185–192. 10.1111/j.1365-2214.2006.00601.x 16441853

[CIT0026] Milbrath, V.M., Cecagno, D., Soares, D.C., Amestoy, S.C. & Siqueira, H.C.H.D., 2008, ‘Being a woman, mother to a child with cerebral palsy’, *Acta Paulista de Enfermagem* 21(3), 427–431. 10.1590/S0103-21002008000300007

[CIT0027] Nimbalkar, S., Raithatha, S., Shah, R. & PanchaL, D.A., 2014, ‘A qualitative study of psychosocial problems among parents of children with cerebral palsy attending two tertiary care hospitals in Western India’, *ISRN Family Medicine* 2014, 769619. 10.1155/2014/769619 24967331 PMC4041266

[CIT0028] Paulson, A. & Vargus-adams, J., 2017, ‘Overview of four functional classification systems commonly used in cerebral palsy’, *Children (Basel)* 4(4), 30. 10.3390/children4040030 28441773 PMC5406689

[CIT0029] Pfeifer, L.I., Silva, D.B.R., Lopes, P.B., Matsukura, T.S., Santos, J.L.F. & Pinto, M.P.P., 2014, ‘Social support provided to caregivers of children with cerebral palsy’, *Child: Care, Health and Development* 40(3), 363–369. 23734935 10.1111/cch.12077

[CIT0030] Phumudzo, R., ShirindI, M.L. & Makofane, M.D.M., 2021, ‘Mothers caring for children living with cerebral palsy: Suggestions for psycho-social support’, *Social Work* 57(3), 359–375. 10.15270/57-3-952

[CIT0031] Pizano-Vega, S., Leichty, J., Peterson, M. & Abraham, S., 2020, ‘Respite care in families of children with disabilities: A literature review’, *International Journal of Science and Research Methadology* 17(2), 96–105.

[CIT0032] Pretorius, C. & Steadman, J., 2018, ‘Barriers and facilitators to caring for a child with cerebral palsy in rural communities of the Western Cape, South Africa’, *Child Care in Practice* 24(4), 413–430. 10.1080/13575279.2017.1347146

[CIT0033] Prieto, V., 2020, ‘Caregivers of medically fragile children with technology needs’, Doctoral dissertation, University of Texas Health Science Center at Houston, School of Nursing, viewed n.d., from https://digitalcommons.library.tmc.edu/uthson_etd/44/?utm_source.

[CIT0034] Proctor, 2022, *Types of cerebral palsy*, viewed 15 January 2023, from https://www.cerebralpalsyguide.com/cerebral-palsy/types/spastic/.

[CIT0035] Rachamose, N. & Harvey, C., 2024, ‘The mental health of maternal caregivers of children with cerebral palsy in rural, low-income parts of Southern Africa’, *African Journal of Social Work* 14(4), 217–224. 10.4314/ajsw.v14i4.5

[CIT0036] Rachamose, N. & Harvey, C., 2025, ‘The barriers to caring for a child living with Cerebral Palsy (CP) in rural Limpopo, South Africa’, *Disabilities* 5(1), 11. 10.3390/disabilities5010011

[CIT0037] Raphulu, P., Shirindi, M.L. & Makofane, M.D.M., 2021, ‘Mothers caring for children living with cerebral palsy: Suggestions for psycho-social support’, *Social Work/Maatskaplike Werk* 57(3), 359–375. 10.15270/52-2-952

[CIT0038] Saloojee, G. & Bezuidenhout, M., 2020, ‘Community-based peer supporters for persons with disabilities: Experiences from two training programmes’, *South African Health Review* 2020, 89–97.

[CIT0039] Singogo, C., Mweshi, M. & Rhoda, A., 2015, ‘Challenges experienced by mothers caring for children with cerebral palsy in Zambia’, *South African Journal of Physiotherapy* 71(1), 274. 10.4102/sajp.v71i1.274 30135879 PMC6093109

[CIT0040] Smith, M. & Blamires, J., 2022, ‘Mothers’ experience of having a child with cerebral palsy. A systematic review’, *Journal of Pediatric Nursing* 64, 64–73. 35158294 10.1016/j.pedn.2022.01.014

[CIT0041] Sodi, T. & Kgopa, B., 2016, ‘Coping strategies of mother carers of children living with chronic illness and disease in a rural South African community’, *Journal of Psychology in Africa* 26, 432–435. 10.1080/14330237.2016.1219546

[CIT0042] Stevenson, A. (ed.), 2010, *Oxford Dictionary of English*, 3rd edn., Oxford University Press, Oxford.

[CIT0043] Thrush, A. & Hyder, A.A., 2014, ‘The neglected burden of caregiving in low- and middle-income countries’, *Disability and Health Journal* 7(3), 262–272. 10.1016/j.dhjo.2014.01.003 24947567

